# Exposure to Community Violence, Psychopathology, and Personality Traits in Russian Youth

**DOI:** 10.1155/2011/909076

**Published:** 2011-08-04

**Authors:** Roman Koposov, Vladislav Ruchkin

**Affiliations:** ^1^RKBU-North, Faculty of Health Sciences, University of Tromsø, Gimlevegen 78, 9037 Tromsø, Norway; ^2^Department of Social and Forensic Psychiatry, Division of Clinical Neuroscience, Karolinska Institute, Box 4044, 14104 Huddinge, Sweden; ^3^Forensic Psychiatric Clinic Sater, Box 350, 78327 Sater, Sweden; ^4^Yale Child Study Center, Yale University School of Medicine, 230 South Frontage Road, New Haven, CT 06520, USA

## Abstract

Previous research with the US inner-city youth demonstrated the hazardous effects of community violence exposure. It remains unclear, however, whether these findings are generalizable to other cultures and populations. Furthermore, the role of factors influencing the processing of traumatic events such as personality has not been investigated. Two groups of Russian adolescents (community youth (*N* = 546) and male delinquents (*N* = 352)) completed questionnaires assessing their exposure to community violence, conduct problems, internalizing psychopathology and personality. The study demonstrates that the relationships between exposure to violence and psychopathology are similar across different populations within the same culture (community youth and juvenile delinquents), suggesting similar mechanisms behind this phenomenon. The patterns of these relationships were also similar for boys and girls, suggesting similarities in the mechanisms across gender. Hence, the effects of community violence exposure are generalizable to other cultures outside the US. The associations between personality traits and specific types of behaviors also tend to be similar across different populations. Higher levels of novelty seeking were related to more severe problem behaviors and to higher levels of witnessing and victimization, whereas higher levels of harm avoidance were related to higher levels of depression and posttraumatic stress.

## 1. Introduction

Research on exposure to community violence, which in early 1990s was called “a public health problem of epidemic proportions” [1], has consistently demonstrated its multiple effects on child and adolescent mental health. These effects include a wide range of internalizing psychopathology, such as posttraumatic stress [2–4], anxiety, and depression [5–8], and of externalizing problems, such as aggressive and delinquent behavior [7–12] and alcohol and drug use [7, 13]. Children who have been exposed to high levels of community violence often have a decreased self-esteem [5], pessimistic view of the future [7, 14], problems with social relationships [1], and poor academic performance [7, 15]. Although the levels of distress caused by traumatic events tend to decrease over time, there is some evidence that violence exposure may have a long-lasting impact on behavior and mental health of children [10, 11].

Although the above-mentioned effects have been reliably assessed and tend to be consistent in different studies, several important considerations should be kept in mind when assessing the relationships between violence exposure and psychopathology. First, there has been only one study outside the USA in Canada [16] and none outside North America that reports on the effects of community violence exposure. It remains unclear whether effects of violence exposure in other countries are similar to those reported in American inner city youth, who often experience higher levels of community violence than youths from other communities, and for whom exposure to violence has become an everyday reality and a source of chronic distress.

Second, it is unclear whether the relationships between violence exposure and psychopathology are different in different populations within the same culture. Recent research, for example, has documented that juvenile delinquents represent a highly traumatized group, with rates of posttraumatic stress approaching 30% [17, 18], related to various traumatic events, including domestic [18] and community violence [17]. Furthermore, the levels of psychopathology in antisocial youth tend to be higher than those in the general population as discussed by Ulzen and Hamilton [19]. Thus, it may be reasonable to suggest that the psychopathological outcomes in delinquent youth may not only be related to the magnitude of exposure, but also involve different mechanisms for its development than in the youth from general population.

Third, youth may report higher levels of exposure to violence, because of their own involvement in violence or in other severe problem behaviors [10]. It is unclear whether the effects of exposure to community violence on internalizing psychopathology are similar for a perpetrator and for an innocent bystander, and, thus, the levels of own involvement in severe problem behaviors should be controlled for when assessing these relationships. This is especially true in a cross-sectional study design when it is impossible to control for a baseline level of problem behaviors. 

In addition, controlling for involvement in severe problem behaviors is important because, as mentioned previously, antisocial youth generally tend to have higher rates of psychopathology compared to their well-adjusted peers [20] and juvenile delinquency has been found associated with high levels of depression, hopelessness, anxiety, and posttraumatic stress [17, 18]. Thus, to demonstrate the relationships between violence exposure and internalizing psychopathology in a more clear-cut fashion, youth's involvement in severe problem behaviors should be controlled for.

Fourth, the effects of violence exposure may, in certain respects, be gender specific. It has been found that although males typically are more likely to experience traumatic events [21, 22], females exposed to trauma are more likely to be diagnosed as having posttraumatic stress [21, 23, 24] or at least to report more posttraumatic stress symptoms [2, 12]. These findings raise a question about the necessity of separate analyses of the relationships for boys and girls, which rarely have been done in the past.

Finally, there is increasing evidence that certain cognitive strategies and related personality functions are involved in the processing of traumatic events [17, 25]. There are numerous studies demonstrating that specific personality traits are associated with certain types of psychopathology [26–28] and that temperament can affect the way in which the consequences of traumatic experiences unfold [29]. Previously, we suggested that increased exploratory activity may predispose an individual to greater violence exposure, whereas higher behavioral inhibition at the same time (and possibly, in the same subject) could lead to higher rates of psychopathology [17]. Clarifying the role of personality functions in the processing of traumatic events might help to develop effective prevention and intervention strategies and could increase an awareness of individual characteristics in the development of traumatic response.

Based on the above-mentioned considerations, we propose to assess the relationships between exposure to community violence and psychopathology, controlling for the levels of involvement in severe problem behavior in two samples of youth. First, we will check, whether the findings from the US inner city populations are applicable to the Russian youths from the general population, with results reported separately for boys and girls. The relationships between violence exposure and internalizing psychopathology will be assessed controlling for levels of severe problem behaviors. We will further assess whether the effects of community violence exposure would show a similar pattern in a group of incarcerated juvenile delinquents from the same geographic area. This group was selected as a population at risk that has been repeatedly exposed to high levels of violence in the past [17, 18]. Finally, we will assess the impact of the temperament traits of novelty seeking and harm avoidance that, after being added to the model, are expected to have moderating effects on the relationships between community violence exposure and psychopathology. These relationships will be assessed in both community and delinquent samples.

To achieve these goals, we will use structural equation modeling and will run several models: (a) a model of relationships between violence exposure and psychopathology, in which we control for levels of severe problem behaviors, first in the general population and second in the delinquent population; (b) a model of relationships between violence exposure and psychopathology with personality traits as moderators, controlling for the levels of severe problem behaviors.

We expected that, similar to the US samples, we would obtain significant relationships between the measures of violence exposure and psychopathology, which will remain significant even after controlling for the levels of severe problem behaviors. We also proposed that these relationships would be moderated by the temperament traits of novelty seeking and harm avoidance, with high novelty seeking related to more externalizing, and high harm avoidance to more internalizing problems. In spite of large potential differences in the levels of exposure to community violence and psychopathology, these relationships are expected to be similar across the three study groups (boys and girls from the community sample, and delinquents).

## 2. Materials and Methods

The study was approved by the appropriate Ethical Committees, including the Institutional Review Board of the Northern State Medical University (Arkhangelsk, Russia).

### 2.1. Community Sample

In this study, which represents a part of an ongoing cross-cultural project that assesses risk and protective factors for adolescent adjustment, surveys were administered to a community sample of 14–18-year-old adolescents (mean age = 15.5 ± 0.9) in a large region in the north of European Russia. The population of the region is very homogeneous, with approximately 98% being ethnic Russian. The socioeconomic status of the majority of the population is estimated to be similar to the (low) Russian average, and interindividual differences in socioeconomic status are minimal. The schools for the assessment were randomly selected from the list of schools in four districts of the city. The assessment was conducted in classes, which were also randomly selected from the list of the classes within each school. A total of 546 subjects were eligible for analyses (189 (34.6%) boys).

### 2.2. Delinquent Sample

Delinquent subjects were recruited voluntarily from a group of male adolescent inmates ages 14–19 years (mean age = 16.4 ± 0.9), who had been court ordered after trial to the only correctional facility for juveniles in the region in the same part of Northern Russia, a catchment area with a population of 1.5 million. Most of the participants had multiple convictions that included property crimes (theft, car theft, and so on—51%), violence-related crimes (e.g., assault, robbery—38%), and, in some cases, rape/sexual violence (6%) or murder (5%). Generally, those institutionalized for theft had shown a repetitive pattern of stealing, with multiple convictions, with sentencing to the correctional facility occurring only after repeated convictions during parole. At the time of the study, the mean length of sentence was 4.3 years and all participants had been incarcerated for at least 6 months. The data were collected in a sample of 352 delinquent youths.

Ethnic minorities in the study group represented less than 1%, with the majority of the sample represented by ethnic Russians. Of the delinquent sample, 120 youth (34.1%) came from a single-parent family, as compared to 80 girls (22.4%) and 36 boys (19.0%) from the general population (Chi-square = 19.23; *P* < .000).

### 2.3. Procedure

The translation of these scales into Russian followed established guidelines, including appropriate use of independent back translations [30]. The translations were made by a working group in Russia, followed by discussion of the translated questionnaires with colleagues. Finally, an independent interpreter made back translations, which were compared with the originals, and inconsistencies were analyzed and corrected. All questionnaires were also pretested in different samples of youths.

In the community sample, both students and their parents were provided with detailed descriptive information about the study and informed of the planned date of the survey administration and parents were informed of their option to decline participation of their child/children. Students also had the option to decline at the time the survey was administered (parents and student refusals <1%). All participants from the delinquent group were similarly informed about the voluntary and confidential nature of their participation in the study. They were further assured that the institutional staff would not obtain any individualized information about the subjects' responses. Questions that arose were answered in detail. Eight delinquent subjects refused to participate because of unwillingness to provide any personal information. 

In both study samples, the survey was completed in 45-minute sessions during a regular school day with the whole class present (generally 25–30 youths at a time). Those students who refused to complete the survey were given alternative tasks. Trained administrators read questions aloud while participants followed along with their copies of the survey, reading questions to themselves and marking responses in the booklets. The administrators also ensured the students privacy while responding.

### 2.4. Instruments

#### 2.4.1. Social and Health Assessment

The Social and Health Assessment, developed by Weissberg et al. [33] and adapted by Schwab-Stone et al. [8], served as the basis for the survey. As described in more detail below, this survey includes several scales available from the literature that have been used with similar populations both in the USA and in other countries.

#### 2.4.2. Violence Exposure

Items from this scale were derived from the Screening Survey of Exposure to Community Violence developed by Richters and Martinez [6]. Using yes/no response format, students were asked whether they had ever witnessed or been victimized by 6 types of violence (been beaten up or mugged, threatened with serious physical harm, shot or shot at with a gun, attacked or stabbed with a knife, chased by gangs or individuals, or seriously wounded in an incident of violence), providing separate scores for witnessing and victimization. The internal consistency coefficients (Cronbach' *α*) for this scale were .67 for witnessing and .46 for victimization in the general population sample and .74 for witnessing and .61 for victimization in the delinquent sample. Low alphas obtained for the indexes of community violence exposure should not be discouraging, as it is inappropriate to expect that life-event lists should display high internal consistency [34]. Indeed, these measures represent coefficients, rather than scales, where witnessing of or victimization by one type of violence does not necessarily imply the presence of another type of exposure. 

#### 2.4.3. Severe Conduct Problems

Eight items describing different types of severe conduct problems (starting a fistfight; participating in gang fights; hurting someone badly in a fight; carrying a gun; having been arrested by police; carrying a blade, knife, or gun in school; suspension from school; being high at school from drinking alcohol or smoking marijuana) were adapted from Jessor et al. [31], NASHS survey [32], or developed specifically for the survey [33]. The respondents were asked to report on a 5-point scale how many times (if any) (ranging from 0 times to 5 or more times) they were involved in the above-mentioned behaviors during the past two-years (in delinquent population, during two year period prior to incarceration). The scale provides a total score that can range from 0 to 40. This scale had a Cronbach' *α* value of .75 in a general population sample and .82 in the delinquent sample.

#### 2.4.4. Psychopathology

To assess psychopathology, two measures were used in the present study. *Child PTSD Reaction Index (CPTSD-RI) *is a 20-item scale designed to assess posttraumatic stress reactions of school-aged children and adolescents after exposure to a broad range of traumatic events [4, 35]. The instrument has a Likert type five-point rating scale ranging from “none” (0) to “most of the time” (4) to rate the frequency of symptoms. Degree of reactions ranges from doubtful to very severe. The scale is highly correlated with the DSM-based diagnosis of posttraumatic stress syndrome [35]. In the present study, an adequate Cronbach' *α* for the scale was obtained for both samples (.81 in the community sample and  .84 in the delinquent sample). The *Beck Depression Inventory* [36] is a 21-item self-report measure that assesses current symptoms of depression. Each item includes four self-evaluative statements that are scored from 0 to 3. The BDI has been found to correlate with psychiatric ratings of depression [37, 38]. Cutoff scores have been established, ranging from minimal to severe depression [37]. In our sample, a good internal consistency for the scale was obtained for both samples (Cronbach' *α* = .86 in the community sample and .87 in the delinquent sample).

#### 2.4.5. TCI (Temperament and Character Inventory [26])

This inventory is based on Cloninger's unified biosocial theory of personality [39] and measures four temperament and three character dimensions. According to Cloninger's theory, temperament dimensions are independent and largely genetically determined [26]. Two scales for temperament related to the study hypothesis were used in the current study (harm avoidance and novelty seeking). Harm avoidance reflects a heritable bias in the inhibition or cessation of behaviors. Subjects scoring high on harm avoidance are pessimistic, chronically worried, shy with strangers, and tense in unfamiliar situations. Novelty seeking is viewed as a tendency toward behavior activation in response to novel stimuli or cues. Subjects high on novelty seeking show high levels of exploratory behavior, impulsive decision making, quick loss of temper, and active avoidance of frustration. 

Cloninger's theory of personality and the TCI have been utilized and validated with adolescents, both in the USA [40] and other cultures [41, 42], including Russia [28]. In the present study, we used the short version of the TCI with 125 items to be answered as true or false. Cronbach' *α*'s for novelty seeking were .63 in the community sample and .60 in delinquents, and for harm avoidance .78 in the community sample and .68 in delinquents.

### 2.5. Data Analysis

The data were analyzed using the Statistical Package for Social Sciences (SPSS-15.0), with the Analysis of Moment Structures [43] used to build a structural equation model. Missing data on the scales (less than 5%) were imputed using a series mean value.

## 3. Results

As presented in Table 1, both boys and girls from a Russian community sample reported relatively high levels of witnessing and victimization, with a general tendency for boys to have higher rates of violence exposure. Delinquent participants reported the highest rates of community violence exposure, which were significantly higher than those in the community sample.

Although girls were less frequently exposed to community violence, they reported higher levels of psychopathology than boys (Table 2), including both depression and posttraumatic stress. The highest levels of psychopathology reported by delinquents were presumably related to their higher levels of traumatization. Predictably, delinquents reported the highest levels of severe problem behaviors, whereas girls in the community sample reported the lowest levels.

As predicted, the levels of severe problem behaviors in both community and delinquent samples were significantly related to witnessing and victimization (Table 3), implying that those involved in antisocial behavior generally would have had more chances to witness community violence or to be victimized by it [44]. In both samples, community violence exposure scores were also significantly related to the scores of psychopathology. Finally, the temperament trait of novelty seeking was significantly related to higher levels of community violence exposure and to higher levels of severe behavior problems, whereas higher levels of harm avoidance were significantly related to higher levels of internalizing psychopathology, and to lower levels of severe problem behaviors (Table 3).

To investigate links between the variables of interest within a model, structural equation modeling techniques were applied. As proposed, two models were tested: (1) the violence exposure-psychopathology model, controlling for the levels of severe problem behaviors and (2) the violence exposure-psychopathology model with novelty seeking and harm avoidance as moderators, controlling for the levels of severe problem behaviors. 

To balance the models, for each scale, except for witnessing and victimization, three subscores were computed based on the item-total correlations within each scale. These subscores were used as manifest variables to produce the latent constructs of severe problem behaviors, depression, posttraumatic stress, and temperament traits of novelty seeking and harm avoidance (for a detailed theoretical explanation of the procedure, see Kishton and Widaman [45] and Little et al. [46]). This procedure was not applied to the scores for witnessing and victimization because they were considered to be coefficients rather than scales, where one type of violence exposure does not necessarily imply the presence of another type. 

**Figure 1:**
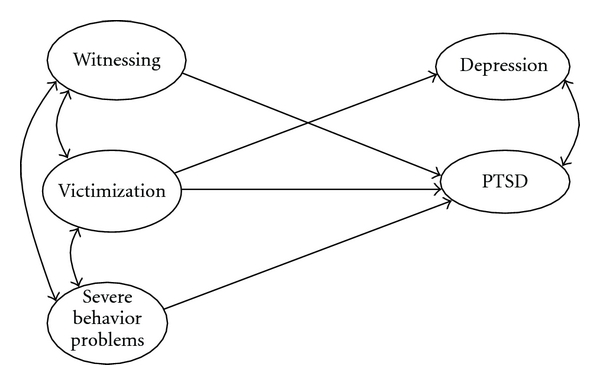
Relationships between violence exposure and psychopathology, with significant paths only (Model 1).

**Figure 2:**
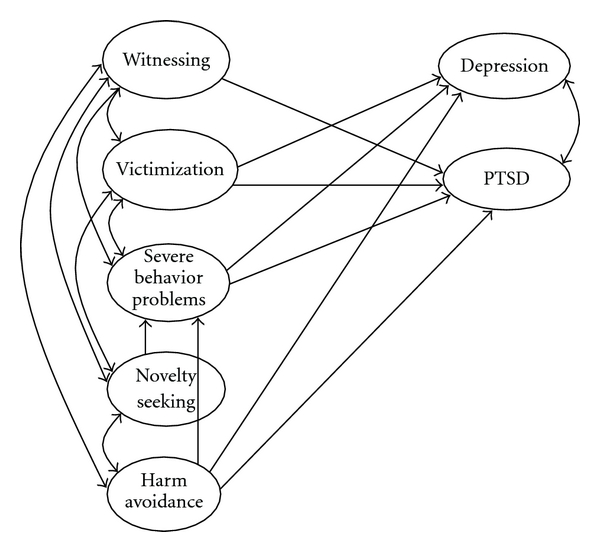
Relationships between violence exposure, personality, and psychopathology, with significant paths only (Model 2).

Model fit was assessed using two standard fit indexes, namely, the root mean squared error of approximation (RMSEA), for which values of .08 or less are deemed acceptable, and the comparative fit index (CFI), for which values greater than .90 are deemed acceptable [42, 47, 48]. Because the maximum likelihood Chi-squared value is highly sensitive to sample size, it was not employed to evaluate overall model fit. The models and model parameters are presented in Figures 1 and 2; the fit statistics for all models is presented in Table 4. 

First, the initial model of violence exposure-psychopathology relationships, controlling for the levels of severe problem behaviors, was assessed in a sample of Russian youths from the general population, separately for boys and girls. A good fit for the model was obtained (*χ* ^2^(82) = 231.3; RMSEA = .058 (.049; .067); CFI = .92). Subsequently, all nonsignificant paths were excluded from the model and the fit of the reduced model (Figure 1) was assessed. The fit for the final (reduced) model is presented in Table 4. Subsequently, the same model was applied to the sample of juvenile delinquents and an even better fit was obtained (*χ* ^2^(37) = 51.2; RMSEA = .033 (.000; .053); CFI = .99). 

All significant relationships (beta weights and SE) and covariates for the Model 1 are presented in Table 4. The findings can be summarized in that, in all three groups, witnessing was related only to posttraumatic stress and victimization, was related to both posttraumatic stress and depression. Also, the scores for posttraumatic stress and depression in all groups were interrelated, suggesting a high degree of comorbidity between these two conditions, as were the scores of witnessing, victimization and severe problem behaviors. The only difference between the models was in the relationship between severe conduct problems and posttraumatic stress, which was positive in girls, nonsignificant in boys, and negative in delinquents. All models had good fit statistics (Table 4).

As a second step, we sought to assess the effects produced by the temperament traits of novelty seeking and harm avoidance, which were expected to have moderating effects on the relationships between community violence exposure and psychopathology. As in Model 1, these relationships were similarly assessed in the community and then in delinquent samples. A good fit for both models was obtained (*χ* ^2^(216) = 274.0; RMSEA = .037 (.030; .043); CFI = .94—for the community sample and *χ* ^2^(101) = 164.0; RMSEA = .042 (.030; .054); CFI = .97—for delinquents). After that, all nonsignificant paths were excluded from the model and the fit of the reduced model (Figure 2) was assessed. The fit for the final Model 2 and all significant relationships (beta weights and SE) and covariates are presented in Table 5. Adding temperament traits in the model did not impact on the relationships between violence exposure and severe problem behaviors or between posttraumatic stress and depression. The relationships between severe problem behaviors and posttraumatic stress, however, became significant and positive in all three groups.

The pattern of relationships between violence exposure scores and psychopathology after introducing temperament traits into the model remained generally the same as in the initial models, although the relationships became somewhat less pronounced. The relationships between novelty seeking, harm avoidance, and psychopathology were similar to those predicted. Higher levels of harm avoidance were related to higher levels of depression and posttraumatic stress, and in some cases were negatively related to the involvement in severe problem behaviors (delinquents) or to witnessing (control boys). Higher levels of novelty seeking in all three samples were related to greater involvement in severe problem behaviors and to higher levels of witnessing and victimization.

## 4. Discussion

The purpose of the present study was to test the model of relationships between exposure to community violence and psychopathology in a community sample of Russian youths, controlling for the involvement in severe problem behaviors, and to further verify this model on a sample of incarcerated juvenile delinquents from the same area. We also sought to investigate whether personality traits would play a moderating role in the relationships between violence exposure and psychopathology and would help to clarify the dynamics of these interactions. 

The novelty of this study is its cross-cultural application of findings that have been to date reported almost exclusively in the USA inner city populations. This study demonstrates that, even in the communities with less pronounced levels of community violence, the effects of violence exposure are still meaningful and related to increased levels of psychopathology. This study also addresses the issue of cross-cultural applicability of the findings reported in the USA, and calls for more attention to this problem from policy makers and mental health professionals in other countries.

This study demonstrates that the trends for the relationships between exposure to violence and psychopathology are also similar across different populations within the same culture, such as youth from a general population and incarcerated juvenile delinquents, suggesting at least some similarities in the mechanisms that underlie this phenomenon in different groups. Similar to the previous studies [21, 22, 24], boys reported more exposure to violence whereas girls reported higher levels of psychopathology. However, the patterns of the relationships between violence exposure and psychopathology for boys and girls were similar, suggesting possible similarities in the underlying mechanisms across gender. Juvenile delinquents reported the highest levels of psychopathology of all three groups. These findings support previous reports suggesting that juvenile delinquents as a population are frequently exposed to various types of violence with various psychopathological manifestations associated with such exposure [17, 18].

As previously suggested by Gorman-Smith and Tolan [10] when considering the relationships between violence exposure and psychopathology, it is important to discriminate between the rates of violence exposure reported by “innocent bystanders” and the rates of violence that might be reported due to own involvement in violence. Those who are involved in antisocial behaviors clearly have more chances to witness violence, or even to be victimized, and this association might distort the “real” relationships between violence exposure and psychopathology. In the present study even after controlling for the levels of severe problem behaviors, the relationships between violence exposure and psychopathology remained significant, suggesting that damaging effects of community violence on the mental health of youth can develop independently of involvement in problem behaviors. 

The association between witnessing and psychopathology was generally less pronounced than that for victimization and psychopathology. In this study, victimization was related not only to posttraumatic stress, but also to depression. Such findings are supported by previous studies [8], which have demonstrated that direct victimization has more significant impact on psychopathology than witnessing does. These findings are also supported by the concept of proximity to trauma, with higher degree of physical proximity associated with greater distress [49]. Other studies similarly demonstrated that sometimes witnessing might be unrelated (or even negatively related) to depression, which can be explained by desensitization due to chronic exposure to community violence [50]. 

In studies of children's reactions to violence exposure, several individual, family, and community factors have been identified as potential moderators, including age and gender of the child, family structure, school characteristics, and peer relationships [51]. There is also increasing evidence that certain cognitive strategies and related personality functions are involved in the processing of traumatic events [17, 25], that specific personality traits are associated with certain types of psychopathology [26–28], and that temperament can affect the way in which the consequences of traumatic experiences unfold [29]. In our previous work, we suggested that increased exploratory activity may predispose an individual to greater violence exposure whereas higher behavioral inhibition at the same time (and possibly, in the same subject) could lead to higher rates of psychopathology [17]. Thus, it is important to understand the impact of personality characteristics on the relationships between violence exposure and psychopathology, as clarifying the role of personality functions in the processing of traumatic events might help to develop effective prevention and intervention strategies and could increase an awareness of individual characteristics in the development of traumatic response.

## 5. Conclusions

Higher levels of novelty seeking in all three samples were related to greater involvement in severe problem behaviors and to higher levels of witnessing and victimization. Indeed, increased behavior activation (high novelty seeking) may potentially predispose youth to greater exposure to risky and violent situations. It has been found previously that youth who engage in antisocial behavior often have higher novelty seeking [27, 28], thus the current findings may reflect the pathways by which personality factors lead to increased violence exposure, both directly and indirectly through the involvement in severe problem behaviors. 

These findings also indicate a relationship between the temperamental pattern of behavior inhibition and psychopathology, with higher levels of harm avoidance related to higher levels of depression and posttraumatic stress and, in some cases, negatively related to the involvement in severe problem behaviors (delinquents) or to witnessing (control boys). Generally, high harm avoidance reflects the tendency of the individual to be more fearful and cautious (and, thus, less involved in problem behaviors and potentially witnessing less traumatic events), as well as nervous, passive, and having low energy levels. These traits are often combined with poor coping skills, factors that make such youth especially sensitive to stressful life events, and potentially lead to various psychopathological manifestations [26] and internalizing problems in youth [28]. Finally, inhibited temperamental patterns have recently been associated with a physiological pattern of resting right frontal EEG activation in children [52, 53], which in adults appears to be associated with a tendency to respond to stressful events with negative affect or depressive symptomatology [54].

Contrary to expectation, higher novelty seeking does not necessarily imply low harm avoidance and, in the present study, harm avoidance and novelty seeking in the delinquent group were unrelated. These traits can be present in various combinations, as suggested by Cloninger [26, 39] in his typology of personality—high and low, high and high, and so forth. We thus suggest that increased exploratory activity may predispose an individual to greater violence exposure, whereas higher behavioral inhibition at the same time could lead to higher rates of psychopathology. Environmental experiences, and particularly violence exposure, filtered through personality traits, may increase individual vulnerability to stress. Our findings also suggest that a wide range of psychopathology may be related to specific reactivity patterns to environmental stress and emphasize the importance of a focus on personality aspects in the treatment of traumatized delinquent youth.

This work has the usual limitations of cross-sectional studies that preclude the possibility of drawing causal relationships. The study relies on self-report measures and is limited by its retrospective assessment of psychopathology and violence exposure. Finally, although the findings expand the results obtained in the US inner city youth and demonstrate that the relationships between exposure to community violence and psychopathology are generalizable to other cultures, this study is nevertheless limited to youth from the Russian North and additional studies should address this issue in other samples and cultures.

## Figures and Tables

**Table 1:** Prevalence of different types of community violence exposure by sample and by gender *N* (%).

*In the past two years*	General population		Delinquents	Chi-square
I have seen…	Girls	Boys		
Someone else getting beaten up or mugged^b,c^	94 (26.3)	51 (27.1)	189 (54.0)	68.41; *P* < .000
Someone else get threatened with serious physical harm^a,b,c^	78 (21.8)	57 (30.2)	157 (45.0)	43.64; *P* < .000
Someone else get shot or shot at with a gun^b,c^	12 (3.4)	8 (4.2)	57 (16.3)	43.38; *P* < .000
Someone else being attacked or stabbed with a knife^a,b,c^	18 (5.0)	15 (7.9)	92 (26.4)	74.35; *P* < .000
Someone else being chased by gangs or individuals^a,b,c^	38 (10.6)	32 (16.9)	100 (28.6)	37.60; *P* < .000
A seriously wounded person after an incident of violence^a,b,c^	27 (7.6)	33 (17.5)	91 (26.1)	43.18; *P* < .000

I have been…				
Beaten up or mugged^a,b,c^	17 (4.8)	27 (14.3)	133 (37.9)	127.13; *P* < .000
Threatened with serious physical harm by someone^a,b,c^	37 (10.4)	31 (16.4)	147 (42.5)	106.88; *P* < .000
Shot or shot at with a gun^b,c^	3 (.8)	2 (1.1)	34 (9.7)	39.67; *P* < .000
Attacked or stabbed with a knife^b,c^	3 (.8)	4 (2.1)	74 (21.1)	102.35; *P* < .000
Chased by gangs or individuals^b,c^	51 (14.3)	22 (11.6)	82 (23.4)	15.23; *P* < .000
Seriously wounded in an incident of violence^b,c^	—	—	19 (5.4)	30.28; *P* < .000

^a^Significant differences between girls and boys from the community sample; ^b^significant differences between girls from the community sample and delinquent boys; ^c^significant differences between boys from the community and delinquent boys.

**Table 2:** Comparison of the variables used in the models across three groups.

	Controls		Delinquents	
	Girls (*N* = 357)	Boys (*N* = 189)	(*N* = 352)	*F* (df), *P*
Witnessing^b,c^	.75 (1.14)	1.04 (1.39)	1.95 (1.77)	62.76 (2, 895); .000
Victimization^b,c^	.31 (.67)	.46 (.80)	1.39 (1.39)	106.82 (2, 895); .000
Severe problem behaviors^a,b,c^	.67 (2.00)	2.62 (4.21)	10.21 (7.74)	298.93 (2, 891); .000
PTSD^a,b,c^	23.63 (10.25)	18.40 (7.91)	26.48 (12.74)	33.77 (2, 892); .000
Depression^a,b,c^	9.29 (7.95)	5.84 (7.12)	17.59 (11.40)	119.05 (11.40); .000
Novelty seeking^b^	11.31 (3.34)	10.85 (3.23)	11.61 (2.94)	3.52 (2, 895); .030
Harm avoidance^a,b^	9.59 (4.42)	7.88 (3.59)	9.12 (3.69)	11.41 (2, 895); .000

^a^Significant differences between girls and boys from the community sample.

^b^Significant differences between girls from the community sample and delinquent boys.

^c^Significant differences between boys from the community and delinquent boys.

**Table 3:** Correlations between the variables used in the models in general/delinquent populations.

					Delinquents			
Controls		1	2	3	4	5	6	7
1	Witnessing	—	.56**	.42**	.31**	.08	.21**	.00
2	Victimization	.44**	—	.40**	.35**	.26**	.16**	.08
3	SPB	.36**	.37**	—	.20**	−.01	.33**	−.14**
4	PTSD	.23**	.23**	.10*	—	.42**	.10	.31**
5	BDI	.08	.13**	.02	.40**	—	.02	.27**
6	Novelty seeking	.17**	.17**	.17**	.17**	−.01	—	.00
7	Harm avoidance	−.04	−.01	−.15**	.35**	.33**	−.11*	—

**P < .01; *P < .05.

**Table 4:** Relationships between the variables of interest in Model 1.

	Controls		Delinquents
	Girls	Boys	
*Model fit*	*χ* ^2^(84) = 233.4; RMSEA = .057 (.049; .066); CFI = .92		*χ* ^2^(38) = 52.8; RMSEA = .033 (.000; .053); CFI = .99
*Beta-weights (SE); P*			
Witnessing-PTSD	.19 (.07); .002	.18 (.10); .055	.20 (.07); .003
Victimization-depression	.16 (.06); .011	.13 (.10); .201	.34 (.07); .000
Victimization-PTSD	.17 (.07); .006	.24 (.11); .035	.23 (.07); .001
SBP-PTSD	.23 (.08); .001	.02 (.17); .890	.03 (.08); .703
SBP-depression	−.05 (.07); .468	.10 (.13); .438	−.16 (.07); .020
*Covariances (SE); P*			
Witnessing-victimization	.41 (.04); .000	.42 (.06); .000	.56 (.04); .000
Victimization-SBP	.32 (.06); .000	.50 (.09); .000	.43 (.05); .000
Witnessing-SBP	.26 (.06); .000	.53 (.09); .000	.46 (.05); .000
Depression-PTSD	.41 (.06); .000	.57 (.06); .000	.39 (.05); .000

**Table 5:** Relationships between the variables of interest in Model 2.

	Controls		Delinquents
	Girls	Boys	
*Model fit*	*χ* ^2^(222) = 386.6; RMSEA = .037 (.031; .043); CFI = .94		*χ* ^2^(104) = 166.2; RMSEA = .041 (.029; .053); CFI = .97
*Regression weights (SE); P*			
Witnessing-PTSD	.18 (.07); .002	.19 (.10); .029	.19 (.08); .004
Victimization-depression	.10 (.06); .089	.16 (.10); .071	.28 (.07); .000
Victimization-PTSD	.11 (.08); .064	.22 (.11); .019	.17 (.08); .011
SBP-PTSD	.32 (.09); .000	.12 (.06); .270	.12 (.07); .095
SBP-depression	.04 (.07); .582	.13 (.05); .184	−.09 (.06); .215
Harm avoidance-depression	.39 (.07); .000	.25 (.09); .004	.28 (.07); .000
Harm avoidance-PTSD	.43 (.08); .000	.35 (.10); .000	.34 (.08); .000
Harm avoidance-SBP	−.10 (.07); .151	.08 (.21); .379	−.16 (.08); .018
Novelty seeking-SBP	.22 (.09); .009	.91 (.23); .000	.50 (.11); .000
*Covariates (SE); P*			
Witnessing-victimization	.39 (.05); .000	.48 (.06); .000	.56 (.04); .000
Victimization-SBP	.24 (.06); .000	.38 (.16); .018	.39 (.06); .000
Witnessing-SBP	.20 (.06); .001	.41 (.16); .008	.37 (.06); .000
Depression-PTSD	.29 (.07); .000	.49 (.07); .000	.32 (.06); 000
Novelty seeking-victimization	.28 (.06); .000	.39 (.09); .000	.22 (.07); .002
Novelty seeking-witnessing	.23 (.07); .000	.41 (.09); .000	.30 (.07); .000
Novelty seeking-harm avoidance	−.19 (.07); .008	−.27 (.11); .010	−.05 (.08); .516
Harm avoidance-witnessing	.04 (.06); .506	−.19 (.08); .015	−.00 (06); .962
